# Perioperative outcomes of neoadjuvant chemotherapy plus camrelizumab compared with chemotherapy alone and chemoradiotherapy for locally advanced esophageal squamous cell cancer

**DOI:** 10.3389/fimmu.2023.1066527

**Published:** 2023-02-07

**Authors:** Baihua Zhang, Hongbo Zhao, Xun Wu, Lianghui Gong, Desong Yang, Xu Li, Xiaoyan Chen, Jigang Li, Wenxiang Wang, Jie Wu, Qin Xiao

**Affiliations:** ^1^ The Second Department of Thoracic Surgery, Hunan Clinical Medical Research Center of Accurate Diagnosis and Treatment for Esophageal Carcinoma, Hunan Cancer Hospital and The Affiliated Cancer Hospital of Xiangya School of Medicine, Central South University, Changsha, China; ^2^ Department of Thoracic Surgery, National Cancer Center/National Clinical Research Center for Cancer/Cancer Hospital and Shenzhen Hospital, Chinese Academy of Medical Sciences and Peking Union Medical College, Shenzhen, China; ^3^ Department of Pathology, Hunan Cancer Hospital and The Affiliated Cancer Hospital of Xiangya School of Medicine, Central South University, Changsha, China; ^4^ Key Laboratory of Translational Radiation Oncology, Hunan Province, The First Department of Thoracic Radiation Oncology, Hunan Cancer Hospital, The Affiliated Cancer Hospital of Xiangya School of Medicine, Central South University, Changsha, Hunan, China

**Keywords:** neoadjuvant therapy, programmed cell death protein-1 inhibitors, immunotherapy, esophagectomy, neoadjuvant chemoradiotherapy, esophageal squmaous cell carcinoma

## Abstract

**Purpose:**

Neoadjuvant chemoimmunotherapy (nCIT) is becoming a new therapeutic frontier for resectable esophageal squamous cell carcinoma (ESCC); however, crucial details and technical know-how regarding surgical techniques and the perioperative challenges following nCIT remain poorly understood. The study investigated and compared the advantages and disadvantages of esophagectomy following nCIT with neoadjuvant chemotherapy (nCT) and chemoradiotherapy (nCRT).

**Methods:**

We retrospectively analyzed data of patients initially diagnosed with resectable ESCC at clinical stage T2-4N+ and received neoadjuvant therapy followed by esophagectomy at the Hunan Cancer Hospital between October 2014 and February 2021. Patients were divided into three groups according to neoadjuvant treatment: (i) nCIT; (ii) nCT; and (iii) nCRT.

**Results:**

There were 34 patients in the nCIT group, 97 in the nCT group, and 31 in the nCRT group. Compared with nCT, nCIT followed by esophagectomy achieved higher pathological complete response (pCR; 29.0% versus 4.1%, p<0.001) and major pathological response (MPR; 52.9% versus 16.5%, p<0.001) rates, more resected lymph nodes during surgery (25.06 ± 7.62 versus 20.64 ± 9.68, *p*=0.009), less intraoperative blood loss (200.00 ± 73.86 versus 266.49 ± 176.29 mL, *p*=0.035), and comparable results in other perioperative parameters. Compared with nCRT, nCIT achieved similar pCR (29.0% versus 25.8%) and MPR (52.9% versus 51.6%, p=0.862) rates, with significantly more lymph nodes resected during surgery (25.06 ± 7.62 versus 16.94 ± 7.24, p<0.001), shorter operation time (267.79 ± 50.67 versus 306.32 ± 79.92 min, *p*=0.022), less intraoperative blood loss (200.00 ± 73.86 versus 264.53 ± 139.76 mL, *p*=0.022), and fewer ICU admissions after surgery (29.4% versus 80.6%, p<0.001). Regarding perioperative adverse events and complications, no significant statistical differences were detected between the nCIT and the nCT or nCRT groups. The 3-year overall survival rate after nCIT was 73.3%, slightly higher than 46.1% after nCT and 39.7% after nCRT, with no statistically significant differences (p=0.883).

**Conclusions:**

This clinical analysis showed that nCIT is safe and feasible, with satisfactory pCR and MPR rates. Esophagectomy following nCIT has several perioperative advantages over nCT and nCRT, with comparable perioperative morbidity and mortality. The long-term survival benefits after nCIT still requires further investigation.

## Introduction

In 2020, esophageal carcinoma was the seventh most prevalent cancer and sixth leading cause of cancer-related death worldwide (1). The predominant esophageal cancer subtype in Asia is esophageal squamous cell carcinoma (ESCC) (2). ESCC accounts for over 84% of newly diagnosed esophageal cancers annually (3, 4). Surgical resection remains the gold standard for patients with locally advanced resectable ESCC. However, studies have shown that local recurrence and distant metastasis occur in approximately 33% of patients who receive surgery alone (5, 6). Thus, ESCC treatment is challenging and requires a multidisciplinary approach to improve the surgical therapeutic effect in locally advanced resectable disease.

Following the launch of a new era in immunotherapy (including programmed cell death-ligand 1 [PD-L1] and programmed cell death protein-1 [PD-1] inhibitors), further exploration of neoadjuvant immunotherapy alone or combined with chemotherapy or chemoradiotherapy is expected to further improve the therapeutic effect in locally advanced resectable ESCC. In a recent systematic review including 27 phase 2 or 3 clinical trials with 815 patients, the pooled pathological complete response (pCR) rate was 32.4% in ESCC after neoadjuvant chemoimmunotherapy(nCIT), with the pooled incidence of treatment-related severe adverse events of 26.9% (7). Zhu et al. reported that neoadjuvant immunochemoradiotherapy could not improve the pCR rate than neoadjuvant chemoradiotherapy (nCRT) for ESCC, but significantly increased the risk of severe adverse events (8). Another multicenter retrospective study that included 370 ESCC patients showed that the pCR rates of mono-immunotherapy, nCIT, and nCRT plus immunotherapy were 12.1%, 25.5%, and 42.3%, respectively (9). Hence, neoadjuvant PD-1/PD-L1 inhibitors in combination with chemotherapy or chemoradiotherapy are becoming a new therapeutic frontier for resectable ESCC with promising clinical outcomes. However, long-term follow-up are warranted to validate the survival benefits of nCIT or nCRT plus immunotherapy.

Camrelizumab is a PD-1 inhibitor produced in China by Jiangsu Hengrui Pharmaceuticals Co, Ltd. (Lianyungang, China). The ESCORT-1st study showed that first-line camrelizumab plus chemotherapy could achieve better disease control and long-term survival in advanced ESCC than chemotherapy alone (10). Several prospective phase-II clinical trials have also demonstrated that after neoadjuvant chemotherapy plus camrelizumab (nCIT) for ESCC, pCR rates ranged from 24.1% to 42.5%, with major pathological response (MPR) rates of between 45% and 68.8% (11–14). However, these sample sizes were small, and only a few studies reported survival results. Additionally, crucial details and technical know-how regarding the surgical techniques and perioperative challenges following nCIT are still poorly understood.

In the present study, we retrospectively reviewed the perioperative outcomes of esophagectomy following nCIT to compare it with surgery after nCT and nCRT. This study aimed to investigate the potential advantages and disadvantages of esophagectomy after nCIT.

## Patients and methods

### Inclusion and exclusion criteria

This is a retrospective, single-center, observational study. Patients initially diagnosed with resectable ESCC at clinical stages T2-4N+ (American Joint Committee on Cancer, 8^th^ edition) and received neoadjuvant therapy followed by curative-intent surgery between October 2014 and February 2021 at the Hunan Cancer Hospital were recruited. The Eastern Cooperative Oncology Group’s performance status of all patients was 0 or 1. Patients were included on the basis of the following criteria; (1): only squamous cell carcinoma components; (2); thoracic ESCC; (3); patients who received neoadjuvant chemotherapy (nCT), nCIT (only camrelizumab), or nCRT followed by esophagectomy; and (4) the chemotherapy regimens only consisted of paclitaxel and platinum. The exclusion criteria were as follows; (1): pathological non-squamous cell carcinoma components; (2); patients with unresectable primary tumors, more than seven lymph node metastases (N3), or distant metastasis (M1) before neoadjuvant treatment; (3); patients with previous cancer type(s) or other concurrent malignant tumors; (4); patients that received other forms of treatment before surgery; and (5) incomplete medical records.

All clinical data were obtained from medical records and retrospectively analyzed. This study was conducted per the Declaration of Helsinki (as revised in 2013). The Ethics Committee of Hunan Cancer Hospital approved this study (No. 2022097), and patients’ written informed consent was obtained.

### Neoadjuvant treatment modalities

Patients were retrospectively divided into three groups according to the neoadjuvant treatment modality they received; (1): the nCT group, including patients who received one to four cycles of paclitaxel combined with platinum chemotherapy (21 days per cycle); (2); the nCIT group, including patients who received conventional chemotherapy (1–4 cycles of paclitaxel and platinum) and camrelizumab (200 mg) on the first day of each cycle; and (3) the nCRT group, including patients who received concurrent chemotherapy (1–4 cycles of paclitaxel and platinum) and radiotherapy (6-MV X-ray, 39.6–45.0 Gy/1.8–2.0 Gy/f) before esophagectomy.

### Surgery and adjuvant therapy

Generally, patients would receive a tumor re-evaluation within 2 to 6 weeks after the last neoadjuvant treatment cycle. Following multidisciplinary discussion, a curative-intent surgical resection was immediately performed when the tumor was considered operable. Overall, esophagectomy with the stomach as the esophageal substitute and cervical or thoracic anastomosis were performed in all patients, while experienced surgeons regularly conducted a standard 2-field lymphadenectomy. Cervical lymphadenectomy (3-field) was performed only when lymph node metastasis was suspected in the neck region.

Adjuvant treatments were then performed on the basis of pathological tumor stage and each patient’s recovery condition. After multidisciplinary discussion, postoperative chemoradiotherapy or chemotherapy alone might be recommended for patients with ypN+ or palliative resection. In the nCIT group, adjuvant therapy with camrelizumab might be recommended for 1 year after surgery.

### Outcome measures and follow-up

As reported in previous studies (15, 16), pCR was defined as no viable tumor cells in the resected specimen. In contrast, MPR was defined as <10% viable residual tumor cells detected in the specimen. Pathological responses were evaluated independently by two experienced pathologists. Treatment-related adverse events (TRAEs) were graded as per the National Cancer Institute Common Terminology Criteria for Adverse Events (CTCAE) version 5.0. Weight loss at initial diagnosis was defined as weight loss detected within six months before the diagnosis of ESCC. Operation time was calculated from incision to wound closure.

Radiographic evaluations were conducted every 3 months for the first 2 years after surgery, and then every 6 months thereafter. Whenever recurrence was suspected, rebiopsy and/or 18F-FDG positron emission tomography-computed tomography (PET-CT) or both were performed to identify the possible recurrence.

### Statistical analysis

The primary endpoint was the MPR rate, and the secondary endpoints were the pCR, perioperative morbidity, and 3-year OS rates. OS was defined as the time (in months) from surgery to the date of death or the last follow-up. Survival analyses were calculated and compared using Kaplan–Meier curves and the log-rank test.

Differences in clinicopathological features between groups were calculated using the chi-square (*χ* ^2^) test or *t*-test. SPSS software 23.0 (IBM Corp., Armonk, NY, USA) was used to perform all statistical analyses. A *p*-value <0.05 (two-sided) was considered to be statistically significant.

## Results

### Overview of patient cohorts

**Figure 1:**
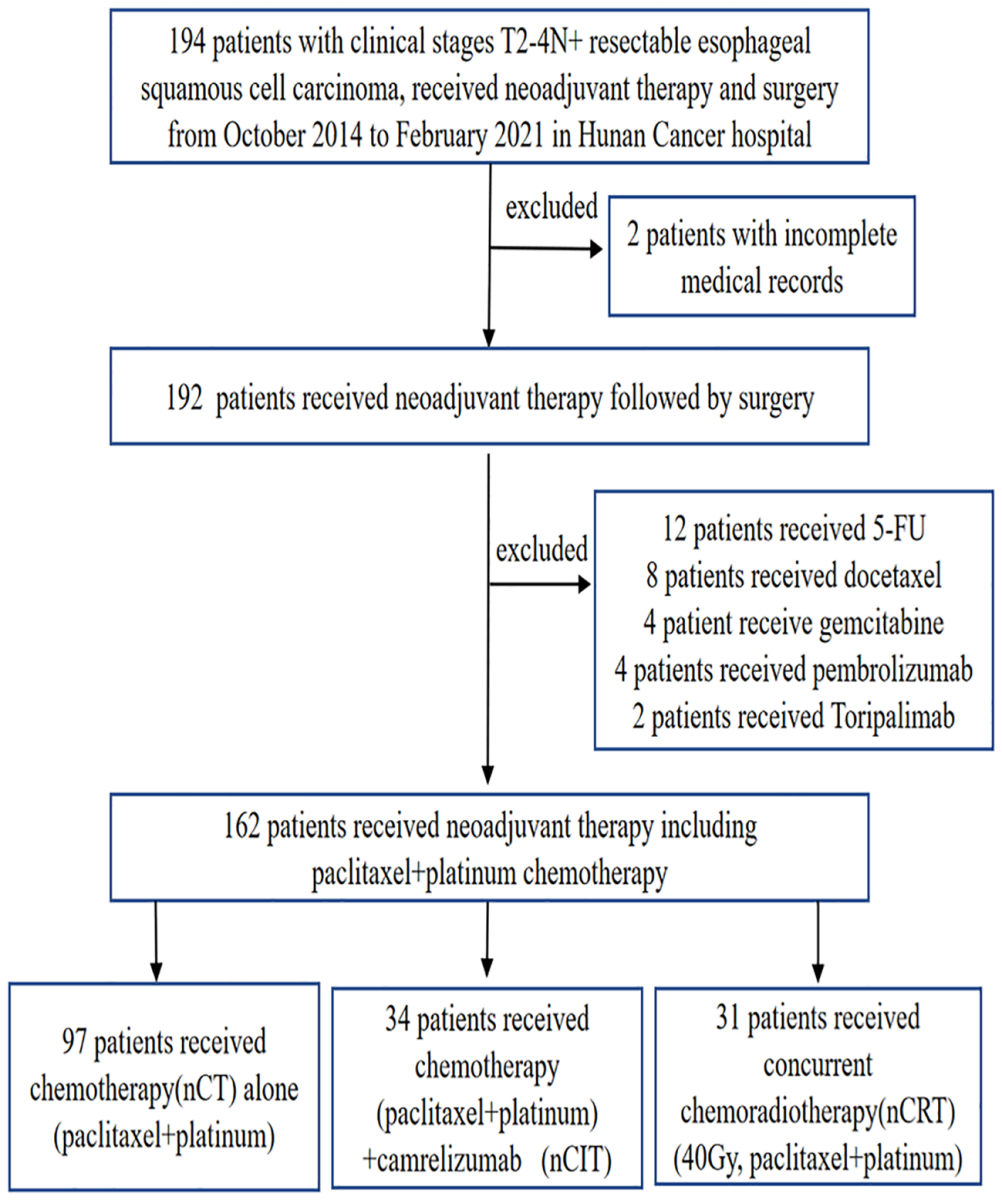
Patient selection flowchart.

Between October 2014 and February 2021, 194 patients were screened for eligibility. Eventually, 162 patients were enrolled for further analysis (34 patients in the nCIT group, 97 in the nCT group, and 31 in the nCRT group) (**Figure 1**). All 162 patients in the study finished one to four cycles of neoadjuvant therapy. As summarized in **Table 1**, the enrolled patients in the nCIT group have a mean age of 60.68 ± 7.44 years old and predominantly consisted of males (91.2%), which were consistent with the nCT and nCRT groups. No significant differences were detected between the nCIT and the nCT or nCRT groups in other baseline characteristics, including cigarette consumption, alcohol abuse, weight loss at initial diagnosis, body mass index, tumor location, tumor length, cN, and pathological differentiation. However, the cT4 percentage in the nCIT group was 35.3%, which was significantly higher than in the nCT group (16.5%, *p*=0.021) but comparable to that in the nCRT group (19.4%, *p*=0.238).

**Table 1:** Clinical characteristics for ESCC patients received neoadjuvant therapy.

Variables	nCIT (n=34)	nCT (n=97)	*P* value	nCIT (n=34)	nCRT (n=31)	*P* value
Age						
Mean ± SD^a^, y	60.68 ± 7.44	60.08 ± 7.78	0.699	60.68 ± 7.44	57.23 ± 6.79	0.056
Gender						
Male	31 (91.2)	94 (96.9)	0.169	31 (91.2)	30 (96.8)	0.348
Female	3 (8.8)	3 (3.1)		3 (8.8)	1 (3.2)	
Cigarette consumption						
Former/current	30 (88.2)	86 (88.7)	0.947	30 (88.2)	27 (87.1)	0.889
No	4 (11.8)	11 (11.3)		4 (11.8)	4 (12.9)	
Alcohol abuse						
Former/current	28 (82.4)	78 (80.4)	0.804	28 (82.4)	29 (93.5)	0.170
No	6 (17.6)	19 (19.6)		6 (17.6)	2 (6.5)	
Weight loss at initial diagnosis						
Yes	21 (61.8)	50 (51.5)	0.303	21 (61.8)	15 (48.4)	0.279
No	13 (38.2)	47 (48.5)		13 (38.2)	16 (51.6)	
BMI index						
Mean ± SD ^a^	21.82 ± 2.74	21.35 ± 3.17	0.442	21.82 ± 2.74	21.77 ± 1.91	0.932
Tumor location						
Upper thoracic	3 (8.8)	10 (10.3)	0.642	3 (8.8)	4 (12.9)	0.169
Middle thoracic	11 (32.4)	39 (40.2)		11 (32.4)	16 (51.6)	
Lower thoracic	20 (58.8)	48 (49.5)		20 (58.8)	11 (35.5)	
Tumor length before treatment						
Mean ± SD ^a^, cm	5.36 ± 1.81	5.11 ± 1.89	0.512	5.36 ± 1.81	5.21 ± 1.72	0.736
cT						
T2/3	22 (64.7)	81 (83.5)	0.021	22 (64.7)	25 (80.6)	0.151
T4	12 (35.3)	16 (16.5)		12 (35.3)	6 (19.4)	
cN						
N1	17 (50.0)	59 (60.8)	0.271	17 (50.0)	11 (35.5)	0.238
N2	17 (50.0)	38 (39.2)		17 (50.0)	20 (64.5)	
Pathological differentiation						
Poor/moderate	27 (79.4)	70 (72.2)	0.407	27 (79.4)	27 (87.1)	0.409
Well	7 (20.6)	27 (27.8)		7 (20.6)	4 (12.9)	

a Variables were described by mean (x) and standard deviation (s).

ESCC, esophageal squamous cell carcinoma; cT, clinical T stage before treatment; cN, clinical N stage before treatment; nCT, neoadjuvant chemotherapy; nCIT, neoadjuvant chemotherapy plus Camrelizumab; nCRT, neoadjuvant chemoradiotherapy.

### Perioperative outcomes

All patients successfully received esophagectomy and most achieved radical resection with no significant statistical differences (**Table 2**). The time interval between final neoadjuvant therapy and surgery in the nCIT group was 35.91 ± 6.76 days, which was significantly longer than in the nCT group (32.70 ± 7.56 days, *p*=0.024) but shorter than in the nCRT group (41.87 ± 10.60 days, *p*=0.010). Patients in the nCIT group (267.79 ± 50.67 min) required a shorter operation time than those in the nCRT group (306.32 ± 79.92 min, *p*=0.022). Meanwhile, no significant difference was detected between the nCIT and nCT groups (291.40 ± 71.48 min, *p*=0.078). Additionally, intraoperative blood loss in the nCIT group (200.00 ± 73.86 mL) was less than in the nCT (266.49 ± 176.29 mL, *p*=0.035) and nCRT (264.53 ± 139.76 mL, *p*=0.022) groups. Notably, 2-field lymphadenectomy was the principal method for lymph node resection in all groups. However, the average number of resected lymph nodes in the nCIT group (25.06 ± 7.62) was significantly higher than in the other two groups (*p*=0.009, *p*<0.001, respectively).

**Table 2:** The perioperative outcomes of esophagectomy after neoadjuvant therapy.

Variables	nCIT (n=34)	nCT (n=97)	P value	nCIT (n=34)	nCRT (n=31)	P value
Interval days						
x ± s ^a^(day)	35.91 ± 6.76	32.70 ± 7.56	0.024	35.91 ± 6.76	41.87 ± 10.60	0.010
Surgical radicality						
Radical	33 (97.1)	89 (91.8)	0.293	33 (97.1)	29 (93.5)	0.500
Palliative	1 (2.9)	8 (8.2)		1 (2.9)	2 (6.5)	
Operation time						
x ± s ^a^(min)	267.79 ± 50.67	291.40 ± 71.48	0.078	267.79 ± 50.67	306.32 ± 79.92	0.022
Intraoperative blood loss						
x ± s ^a^(ml)	200.00 ± 73.86	266.49 ± 176.29	0.035	200.00 ± 73.86	264.53 ± 139.76	0.022
Extent of lymph node resection						
2-field	34 (100.0)	96 (99.0)	0.552	34 (100.0)	30 (96.8)	0.291
3-field	0	1 (1.0)		0	1 (3.2)	
Resected lymph nodes number						
x ± s ^a^	25.06 ± 7.62	20.64 ± 9.68	0.009	25.06 ± 7.62	16.94 ± 7.24	< 0.001
Anastomosis position						
Neck	33 (97.1)	90 (92.8)	0.370	33 (97.1)	26 (83.9)	0.067
Thoracic	1 (2.9)	7 (7.2)		1 (2.9)	5 (16.1)	
Total drainage after operation						
x ± s ^a^(ml)	1925.29 ± 2239.05	2476.25 ± 3335.70	0.285	1925.29 ± 2239.05	3664.35 ± 6581.08	0.151
Duration of chest tube						
x ± s ^a^(day)	8.00 ± 4.70	8.78 ± 3.47	0.378	8.00 ± 4.70	11.42 ± 19.98	0.336
ICU stay after surgery						
Yes	10 (29.4)	31 (32.0)	0.783	10 (29.4)	25 (80.6)	< 0.001
No	24 (70.6)	66 (68.0)		24 (70.6)	6 (19.4)	
Hospital stays after surgery						
x ± s ^a^(day)	12.76 ± 7.30	12.27 ± 4.71	0.713	12.76 ± 7.30	15.65 ± 19.38	0.423
Pathological response						
MPR	18 (52.9)	16 (16.5)	< 0.001	18 (52.9)	16 (51.6)	0.862
PR	9 (26.5)	48 (49.5)		9 (26.5)	7 (22.6)	
SD/PD	7 (20.6)	33 (34.0)		7 (20.6)	8 (25.8)	
ypT stage						
T0-2	23 (67.6)	38 (39.2)	0.004	23 (67.6)	18 (58.1)	0.424
T3-4	11 (32.4)	59 (60.8)		11 (32.4)	13 (41.9)	
ypN stage						
N-	20 (58.8)	45 (46.4)	0.212	20 (58.8)	18 (58.1)	0.951
N+	14 (41.2)	52 (53.6)		14 (41.2)	13 (41.9)	
ypTNM stage						
0-II	22 (64.7)	45 (46.4)	0.179	22 (64.7)	20 (64.5)	0.839
III	9 (26.5)	41 (42.3)		9 (26.5)	7 (22.6)	
IVA	3 (8.8)	11 (11.3)		3 (8.8)	4 (12.9)	
Positive lymph nodes number						
x ± s ^a^	1.32 ± 2.43	1.51 ± 2.36	0.707	1.32 ± 2.43	1.06 ± 1.98	0.642
LVI/perineural invasion						
Yes	6 (17.6)	14 (14.4)	0.654	6 (17.6)	2 (6.5)	0.170
No	28 (82.4)	83 (85.6)		28 (82.4)	29 (93.5)	
Adjuvant therapy						
Yes	20 (58.8)	46 (47.4)	0.253	20 (58.8)	15 (48.4)	0.399
No	14 (41.2)	51 (52.6)		14 (41.2)	16 (51.6)	

a Variabls were described by mean (x) and standard deviation (s).

nCIT, neoadjuvant chemotherapy plus Camrelizumab; nCT, neoadjuvant chemotherapy; nCRT, neoadjuvant chemoradiotherapy; Interval days, interval days between final neoadjuvant therapy and surgery; ypT, pathological T stage after neoadjuvant therapy; ypN, pathological N stage after neoadjuvant therapy; ypTNM, pathological TNM stage after neoadjuvant therapy; ICU, intensive care unit; LVI, lymphovascular invasion; MPR, major pathological response; PR, partial response; SD, stable disease; PD, progressive disease.

Three-incisional esophagectomy with anastomosis in the neck was the principal surgery in all three groups. As summarized in **Table 2**, no significant differences were detected between the groups in the total drainage after operation, duration of chest tube, and hospital stay after surgery. The frequency of ICU stay after surgery in the nCIT group (29.4%) was comparable with that in the nCT group (32.0%, p=0.783) but significantly lower than in the nCRT group (80.6%, p<0.001).

### Pathological efficacy

In the pathological analysis after surgery, MPR was observed in 18 patients in the nCIT group (52.9%), including nine primary tumor pCRs (26.4%) (8 T0N0 [23.5%], 1 T0N+ [2.9%]), and nine patients (26.5%) had 1% to 10% viable residual tumor cells detected in the specimens. In the nCT group, MPR was achieved in 16 patients (16.5%), including four primary tumor pCRs (4.1%) (3 T0N0 [3.1%], 1 T0N+ [1.0%]), which was significantly lower than in the nCIT group (p<0.001). In the nCRT group, 16 patients (51.6%) achieved MPR, including eight primary tumor pCRs (25.8%) (7 T0N0 [22.6%], 1 T0N+ [3.2%]), which was similar to the nCIT group (*p*=0.862).

Accordingly, the ypT0-2 percentage in the nCIT group (67.6%) was also significantly higher than in the nCT group (39.2%, p=0.004) but similar to the nCRT group (58.1%, p=0.424). No significant differences were detected between the groups for other pathological parameters including ypN stage, ypTNM stage, positive lymph node number, and lymphovascular invasion (LVI), or perineural invasion. After surgery, approximately half of the patients received adjuvant therapy, with no statistically significant difference observed among the three groups.

### Perioperative adverse events and complications

The adverse events related to neoadjuvant therapy are summarized in **Table 3**. The frequency of adverse events in the nCIT group was 47.1%, which was comparable with the nCT and nCRT groups. Regarding CTCAE grade, the frequency of severe adverse events (grade III/IV) in the nCIT group was 25.0%, which was similar to the 16.2% and 41.1% in the nCT and nCRT groups, respectively. No deaths related to neoadjuvant therapy (CTCAE grade V) were observed in any group. As to the adverse event types, myelosuppression and erythra were the principal events in the nCIT group, which was different from that of myelosuppression and gastrointestinal react in the nCT group (p=0.002).

**Table 3:** The adverse events of neoadjuvant therapy.

Variables	nCIT (n=34)	nCT (n=97)	*P* value	nCIT (n=34)	nCRT (n=31)	*P* value
Adverse events						
Yes	16 (47.1)	37 (38.1)	0.362	16 (47.1)	17 (54.8)	0.531
No	18 (52.9)	60 (61.9)		18 (52.9)	14 (45.2)	
CTCAE grade						
Any grade	N=16	N=37		N=16	N=17	
I	6 (37.5)	15 (40.5)	0.765	6 (37.5)	4 (23.5)	0.596
II	6 (37.5)	16 (43.2)	0.877	6 (37.5)	6 (35.3)	0.859
III	2 (12.5)	3 (8.1)	0.465	2 (12.5)	4 (23.5)	0.329
IV	2 (12.5)	3 (8.1)	0.465	2 (12.5)	3 (17.6)	0.566
V	0	0		0	0	
Adverse event types						
Myelosuppression	7 (43.8)	18 (48.6)	0.002	7 (43.8)	12 (70.6)	0.129
Erythra	7 (43.8)	1 (2.7)		7 (43.8)	1 (5.9)	
Hepatic dysfunction	1 (6.3)	4 (10.8)		1 (6.3)	1 (5.9)	
Gastrointestinal react	1 (6.3)	12 (32.4)		1 (6.3)	2 (11.8)	
Renal dysfunction	0	2 (5.49)		0	1 (5.9)	

nCT, neoadjuvant chemotherapy; nCIT, neoadjuvant chemotherapy plus Camrelizumab; nCRT, neoadjuvant chemoradiotherapy; CTCAE, Common Terminology Criteria for Adverse Events (version 5.0).

Postoperative complications related to surgery within 30 days occurred in 17 patients (50.0%) in the nCIT group, approximately 37 patients (38.1%) in the nCT group, and 13 patients (41.9%, *p*=0.227) in the nCRT group (*p*=0.515) (**Table 4**). The principal complications included pulmonary complications, anastomotic leakage, hoarseness, and cardiac complications, and these were unrelated to the neoadjuvant therapeutic modality. One patient in the nCIT group received a reoperation within 30 days due to diaphragmatic hernia and chyle, four patients in the nCT group due to anastomotic leakage or tracheostomy, and one patient in the nCRT group due to intrathoracic anastomotic leakage.

**Table 4:** Perioperative complications within 30 days after surgery and mortality.

Variables	nCIT (n=34)	nCT (n=97)	*P* value	nCIT (n=34)	nCRT (n=31)	*P* value
Postoperative complications						
Yes	17 (50.0)	37 (38.1)	0.227	17 (50.0)	13 (41.9)	0.515
No	17 (50.0)	58 (61.9)		17 (50.0)	18 (58.1)	
**Complication types**	n=17	n=37		n=17	n=13	
Hoarseness	2 (11.8)	6 (16.2)		2 (11.8)	1 (7.7)	
Pulmonary complications	7 (41.7)	10 (27.0)		7 (41.7)	3 (23.1)	
Cardiac complications	2 (11.8)	1 (2.7)		2 (11.8)	2 (15.4)	
Chyle	1 (5.9)	3 (8.1)		1 (5.9)	0	
Anastomotic leakage	2 (11.8)	8 (21.6)		2 (11.8)	4 (30.8)	
Gastric and intestinal complications	1 (5.9)	3 (8.1)		1 (5.9)	0	
Other complications	2 (11.8)	6 (16.2)		2 (11.8)	3 (23.1)	
Reoperation in 30 days						
No	33 (97.1)	93 (95.9)	0.757	33 (97.1)	30 (96.8)	0.947
Yes	1 (2.9)	4 (4.1)		1 (2.9)	1 (3.2)	
30-day mortality						
No	34 (100.0)	96 (99.0)	0.552	34 (100.0)	31 (100.0)	1.000
Yes	0	1 (1.0)		0	0	
90-day mortality						
No	31 (91.2)	90 (92.8)	0.761	31 (91.2)	31 (100.0)	0.09
Yes	3 (8.8)	7 (7.2)		3 (8.8)	0	

nCT, neoadjuvant chemotherapy; nCIT, neoadjuvant chemotherapy plus Camrelizumab; nCRT, neoadjuvant chemoradiotherapy.

Only one patient suffered from sudden death, which was 11 days after surgery, and the patient was in the nCT group. The 90-day mortality rate was 8.8% in the nCIT group, and 7.2% in the nCT group (*p*=0.761), while no deaths within 90 days were observed in the nCRT group. No statistically significant difference was found between the nCIT and nCRT groups (*p*=0.09).

### Overall survival and analysis of prognostic factors

**Figure 2:**
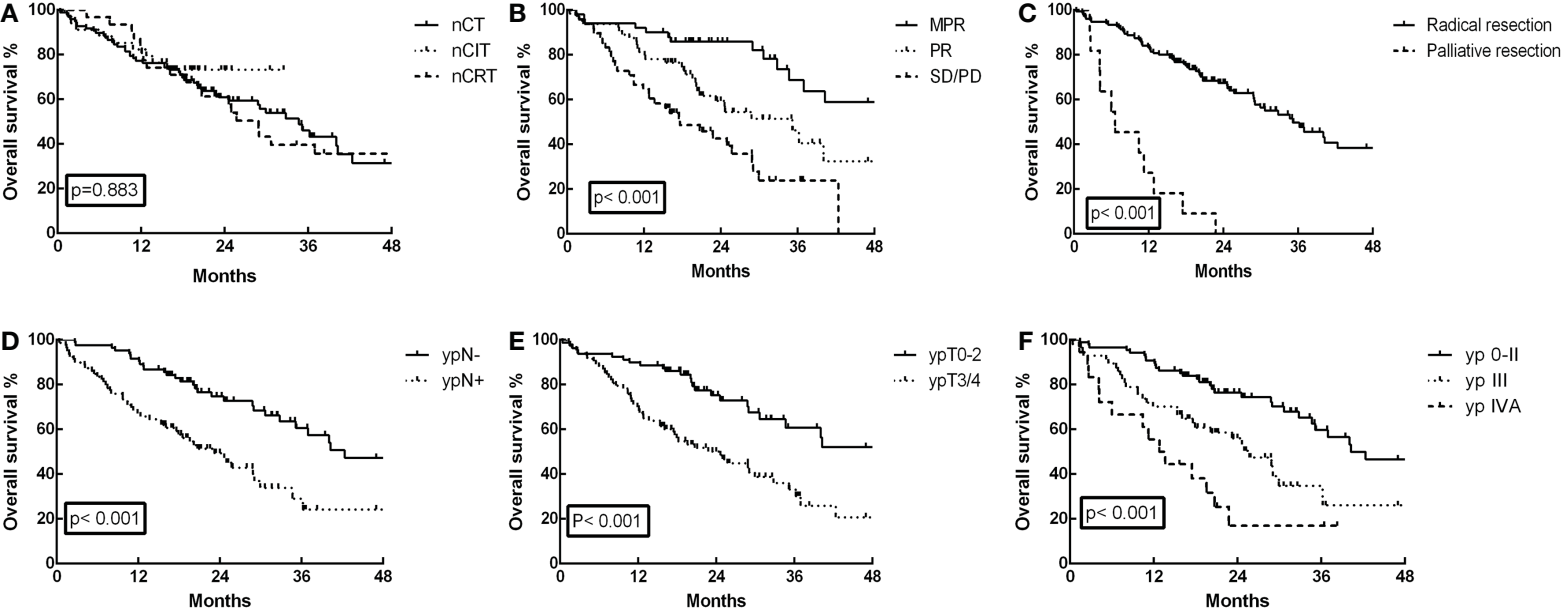
Overall survival (OS) curves for the 162 ESCC patients. **(A)** OS was not significantly different in the neoadjuvant chemoimmunotherapy (nCIT) group compared with the neoadjuvant chemotherapy (nCT) and neoadjuvant chemoradiotherapy (nCRT) groups (p=0.883). **(B)** OS was significantly increased for patients who achieved major pathological response (MPR) compared with those who achieved partial response (PR) and stable disease (SD)/progressive disease (PD) (p<0.001). **(C)** OS was increased for patients who achieved radical esophagectomy compared with those who achieved palliative surgery (p<0.001). **(D)** OS was increased in patients with stage ypN- compared with those who had stage ypN+ (p<0.001). **(E)** OS was increased in patients with stage ypT0-2 compared with those who had stage ypT3/4 (p<0.001). **(F)** OS was increased in patients with stage yp0-II compared with those who had stage ypIII and ypIVA (p<0.001).

Until July 30, 2022, the median follow-up of the entire cohort was 20.45 months, with a range of 0.36 to 84.76 months. In the nCIT group, the 1- and 3-year OS rates were 82.4% and 73.3%, respectively, which were not significantly different from the nCT group (77.3% and 46.1%, respectively) and the nCRT group (83.9% and 39.7%, respectively) (**Figure 2A**, *p*=0.883). Furthermore, the 3-year OS for patients who achieved MPR was 68.7%, which was significantly higher than 46.3% for partial responders and 23.8% for those with stable/progressive disease (**Figure 2B**, *p*<0.001). Patients who achieved radical esophagectomy attained a much better 3-year OS rate than those who achieved palliative surgery (49.7% versus 0%, **Figure 2C**, *p*<0.001). In the analysis of postoperative pathological stage, patients with stage ypN- achieved a 3-year OS of 60.6%, which was longer than the 29.0% for patients with ypN+ (**Figure 2D**, *p*<0.001). Further analysis showed that patients with earlier ypT0-2 and yp0-II staged disease also had better long-term survival rates (**Figures 2E, F**, *p*<0.001).

Interestingly, the 3-year OS rate of patients with weight loss at initial diagnosis was 38.7%, which was significantly shorter than the 55.8% for patients without weight loss (*p*=0.032). Additionally, the 3-year OS for patients without LVI/perineural invasion was 48.3%, which was longer than the 33.8% for patients with LVI/perineural invasion (*p*=0.022). However, age, sex, body mass index, tumor length at initial diagnosis, tumor location, pathological differentiation, and adjuvant systemic therapy were not significantly correlated with OS in univariate Cox analysis (**Table 5**).

**Table 5:** Univariate and multivariate analysis of OS for 162 ESCC patients treated with surgery following neoadjuvant therapy.

Characteristics	Univariate		Multivariate	
	HR (95% CI)	P	HR (95% CI)	P
**Age (y):** < 60 vs ≥ 60	1.039 (0.660-1.634)	0.870		
**Gender:** Male vs Female	0.564 (0.138-2.299)	0.424		
**Weight loss at initial diagnosis:** Yes vs No	1.679 (1.047-2.692)	0.032*		
**BMI index:** < 22 vs ≥ 22	0.870 (0.545-1.390)	0.561		
**Tumor length at initial diagnosis:** <5 vs ≥5cm	0.981 (0.620-1.553)	0.936		
**Tumor location:** Lower vs Upper/middle	1.250 (0.793-1.972)	0.337		
**Pathological differentiation:** Poor/moderate vs Well	1.170 (0.673-2.035)	0.579		
**Surgical radicality:** Palliative vs Radical	7.415 (3.765-14.605)	<0.001*	5.882 (2.799-12.359)	<0.001
**Pathological response:** SD(PD) vs PR vs MPR(CR)	2.090 (1.533-2.849)	<0.001*	1.493 (1.040-2.143)	0.030
**ypT stage:** ypT3-4 vs ypT0-2	2.555 (1.569-4.161)	<0.001*		
**ypN stage:** ypN+ vs ypN-	2.601 (1.615-4.190)	<0.001*	2.100 (1.245-3.542)	0.005
**ypTNM stage:** IVA vs III vs 0-II	1.588 (1.300-1.939)	<0.001*		
LVI/perineural invasion: Yes vs No	2.026 (1.105-3.715)	0.022*		
**Neoadjuvant therapeutic modality:** nCRT vs nCIT vs nCT	0.990 (0.872-1.125)	0.883		
**Adjuvant systemic therapy:** Yes vs No	0.957(0.606-1.511)	0.849		

*Factors included into multivariate analysis.

ESCC, esophageal squamous cell carcinoma; OS, overall survival; nCIT, neoadjuvant chemotherapy plus Camrelizumab; nCT, neoadjuvant chemotherapy; nCRT, neoadjuvant chemoradiotherapy; ypT, pathological T stage after neoadjuvant therapy; ypN, pathological N stage after neoadjuvant therapy; ypTNM, pathological TNM stage after neoadjuvant therapy; vs, versus; HR, hazard ratio; CI, confidence interval.

In the multivariate analysis, which included significant factors identified by univariate analysis, only surgical radicality (hazard ratio [HR]: 5.882, 95% confidence interval [CI]: 2.799–12.359, *p*<0.001), pathological response (HR: 1.493, 95% CI: 1.040–2.143, *p*=0.030), and ypN stage (HR: 2.100, 95% CI: 1.245–3.542, *p*=0.005) were found to be independent prognostic factors for OS other than neoadjuvant modality (**Table 5**).

## Discussion

This study described potential intraoperative technical challenges after nCIT and compared them with other neoadjuvant treatment modalities including nCT and nCRT. Compared with nCT, nCIT followed by esophagectomy achieved higher pCR and MPR rates, more resected lymph nodes during surgery, less intraoperative blood loss, and comparable results in other perioperative parameters. Compared with nCRT, nCIT achieved similar pCR and MPR rates, significantly more resected lymph nodes during surgery, shorter operation time, less intraoperative blood loss, and less frequent ICU stays after surgery. Regarding postoperative complications, no significant statistical difference was detected between the nCIT and the nCT or nCRT groups.

Over the past decade, there have been lingering controversies concerning the effects of neoadjuvant chemotherapy (nCT), chemoradiotherapy (nCRT), and immunotherapy for ESCC. There is still no convincing evidence to prove which neoadjuvant therapeutic modality is best for locally advanced resectable ESCC. Pathological responses including pCR and MPR have been considered as principal surrogate endpoints to evaluate the therapeutic efficacy of different neoadjuvant treatments. Previous large-scale randomized clinical trials have reported that nCRT could achieve higher pCR rates (43.2-49%) than nCT (3.8-10.7%) in ESCC, but nCRT might have more postoperative complications and higher postoperative mortality, with no identified overall survival differences (4, 17–24). Therefore, in Western countries, many institutions have adopted nCRT followed by esophagectomy, but globally, many other countries support nCT alone (4, 25).

In this study, the pCR rate for the primary tumor was 26.4%, and the MPR rate was 52.9% after nCIT, consistent with previous reports (9, 11, 12, 26–30). However, after nCT for ESCC, the pCR rate in previous studies is usually between 3.8% and 10.7% (23, 24, 31), which is close to the 4.1% for the primary tumor in this study and significantly lower than the results for nCIT. In contrast, the pCR rate for nCRT has reached approximately 28.9% to 49% in previous studies, which is slightly better than the 25.8% in this study (21, 24, 31–33). Xu et al. demonstrated that the pCR rate was comparable between nCIT and nCRT (29.8% vs 34.0%), with no significant differences in the incidence of postoperative complications and 30-day mortality (34). Thus, this study showed that ESCC might achieve better therapeutic efficacy from nCIT and nCRT on the basis of pCR and MPR results.

Although the pathological efficacy was better for nCIT and nCRT, controversies concerning the long-term survival results remained. Previous prospective trials on esophageal cancer, including JCOG9907, OEO2, CROSS, and NEOCRTEC5010 have demonstrated that nCT or nCRT can achieve better OS results than surgery alone or postoperative chemotherapy (20, 21, 32, 35). Nonetheless, survival differences between different neoadjuvant therapeutic modalities have not been clarified. Klevebro et al. and Wang et al. showed that nCRT could result in a higher pCR rate than nCT, but with no significant survival benefits (24, 31). Another study showed no significant differences in the 5-year OS or the 5-year relapse-free survival (RFS) rates between nCRT and nCT (36). Two separate meta-analyses also reported that nCRT did not show significant long-term survival benefits as nCT (37, 38). In this study, the 1-year OS rate in the nCIT group were 82.4%, consistent with the 1-year OS of between 87.6% and 92.8% in previous reports (29, 39), but not significantly different from nCT (77.3%) and nCRT (83.9%). In a few propensity score matching analyses, the 1-year OS rate in the nCIT group was 94.5-95.7%, slightly better than 84.8% in the nCT group and 86.2% in the nCRT group, but with no significant statistical differences (40, 41). Although no statistically significant difference was observed in our data, the 3-year OS after nCIT was 73.3%, slightly higher than 46.1% after nCT and 39.7% after nCRT. However, the sample size and follow-up time in the present study were too limited to report mature OS results. Therefore, the survival benefit after nCIT in locally advanced resectable ESCC still requires further investigation. Furthermore, as previously reported (4, 36, 42), our further analysis showed that radical esophagectomy, MPR, and ypN0 (no lymph node metastasis) were independent favorable prognostic factors for OS after neoadjuvant therapy. As to adjuvant therapy, approximately half of the patients received adjuvant therapy in each group, and no statistically significant difference was observed among the three groups. No survival difference was observed between patients received adjuvant therapy or not in our analysis.

This study also highlighted advantages for esophagectomy, as nCIT had more lymph nodes resected and less intraoperative blood loss compared with nCT. During our surgery, tumor and metastatic lymph nodes regression was more significant in the nCIT group than in the nCT group, facilitating lymph node clearance and reducing operation times. Qiao et al. also reported that patients who received nCIT had more lymph nodes cleared during surgery than those who received nCT (34 vs. 30, p<0.001), with comparable incidence of complications (23). Furthermore, when compared with nCRT, esophagectomy after nCIT also achieved more resected lymph nodes, shorter operation times, less intraoperative blood loss, and less frequent ICU stays after surgery. Based on our surgical experience, mild or moderate tissue adhesions were more frequently observed in the nCIT group compared to the nCRT group, which might help reduce the intraoperative difficulties. In certain propensity score matching analyses by Hong et al. and Xiao et al, esophagectomy after nCIT required shorter operative times, and dissected more lymph nodes than after nCRT (41, 43). Cheng et al. also reported that the nCIT group had minimal intraoperative blood loss, shorter operative times, and fewer perioperative complications than the nCRT group (37). However, the extent of lymph node resection and positive lymph node numbers after nCIT were similar to after nCT and nCRT in this study. Regarding other perioperative parameters such as radical resection rate and several postoperative recovery parameters, no significant differences were detected among the three groups.

Perioperative morbidity and mortality are the principal concerns in surgical treatment following neoadjuvant therapy. This study detected no significant statistical differences in the CTCAE grade related to neoadjuvant therapy and postoperative complication types among the three groups. Thus, the addition of camrelizumab to nCT did not increase morbidity or mortality. Additionally, another study by Qiao et al. showed that the total incidence of adverse events during nCIT was lower (77.1%) than nCT (91.7%, *p*=0.003) (23). As reported in previous studies (26, 30), pneumonia was the most prevalent major 30-day postoperative complication in this study. Other common complications included hoarseness, cardiac complication, and anastomotic leakage. Fortunately, no treatment- or surgery-related deaths were observed within 30 days after esophagectomy in this study, except for one sudden death in the nCT group, proving that esophagectomy following nCIT is safe and feasible.

Some limitations are apparent in this study. First, selection biases were inevitable between groups due to the limited sample size and the retrospective design. For example, the cT4 percentage in the nCIT group was 35.3%, which was significantly higher than in the nCT group. Second, the follow-up and recurrences data are insufficient to report mature OS and disease-free survival results. Third, each neoadjuvant therapy might have specific benefits for patient subgroups. The information on predictive biomarkers for therapeutic efficacy, such as PD-L1 expression, was absent in the present study. Therefore, the problem remains with selecting optimal patients who might benefit from different therapeutic modalities. Hence, more prospective phase III clinical trials with larger sample sizes and multiple centers should be conducted to identify the advantages and disadvantages of each neoadjuvant therapy in locally advanced resectable ESCC.

## Conclusion

In conclusion, this real-world analysis showed that nCIT is safe and feasible, with satisfactory pCR and MPR rates. Esophagectomy following nCIT achieved several perioperative advantages over nCT and nCRT, with comparable perioperative morbidity and mortality. Although the 3-year OS after nCIT is slightly higher, the long-term survival benefits still require further investigation.

## Data availability statement

The original contributions presented in the study are included in the article/supplementary material. Further inquiries can be directed to the corresponding authors.

## Ethics statement

The studies involving human participants were reviewed and approved by The Ethics Committee of Hunan Cancer Hospital (No. 2022097). The patients/participants provided their written informed consent to participate in this study.

## Author contributions

(I) Conception and design: BZ, QX, W, JW, XW, HZ. (II) Administrative support: BZ, QX. (III) Provision of study materials or patients: BZ, JW, DY, XL, WW, QX, XW, HZ, LG, XC, JL. (IV) Collection and assembly of data: BZ, JW, QX, XW, HZ, LG. (V) Data analysis and interpretation: BZ, QX. (VI) Manuscript writing: all authors. (VII) Final approval of manuscript: all authors. All authors contributed to the article and approved the submitted version.
